# Laser-Assisted Mist Capillary Self-Alignment

**DOI:** 10.3390/mi8120361

**Published:** 2017-12-15

**Authors:** Bo Chang, Zhaofei Zhu, Mikko Koverola, Quan Zhou

**Affiliations:** 1 College of Mechanical and Electrical Engineering, Shaanxi University of Science and Technology, Xi’an 710021, China; zhuzhaofei@sust.edu.cn; 2 Department of Applied Physics, Aalto University, 02150 Espoo, Finland; 3 Department of Electrical Engineering and Automation, Aalto University, 02150 Espoo, Finland; mikko.koverola@aalto.fi (M.K.); quan.zhou@aalto.fi (Q.Z.)

**Keywords:** mist capillary self-alignment, laser die transfer, hydrophilic/superhydrophobic patterned surfaces, microasssembly

## Abstract

This paper reports a method combining laser die transfer and mist capillary self-alignment. The laser die transfer technique is employed to feed selected microchips from a thermal release tape onto a receiving substrate and mist capillary self-alignment is applied to align the microchips to the predefined receptor sites on the substrate in high-accuracy. The parameters for a low-power laser die transfer process have been investigated and experimentally optimized. The acting forces during the mist-induced capillary self-alignment process have been analyzed and the critical volume enabling capillary self-alignment has been estimated theoretically and experimentally. We have demonstrated that microchips can be transferred onto receptor sites in 300–400 ms using a low-power laser (100 mW), and chips can self-align to the corresponding receptor sites in parallel with alignment accuracy of 1.4 ± 0.8 μm. The proposed technique has great potential in high-throughput and high-accuracy assembly of micro devices. This paper is extended from an early conference paper (MARSS 2017).

## 1. Introduction

High-throughput and high-accuracy microassembly is still a challenging task due to the undesired adhesion forces between micro parts and handling tools at the microscale. To tackle the challenge, microassembly driven by capillary forces [1,2,3,4,5], electrostatic forces [6], and magnetic forces [7] have been reported for various applications, such as assembly of radio frequency identification (RFID) [8], electrical networks [9], lighting emitting diodes [10], and solar cells [11]. Especially, capillary forces have been extensively exploited in fluidic self-assembly [1,11,12], droplet self-assembly [1,13,14], and capillary self-folding [15,16]. In microassembly, micro parts need to be transported to the desired assembly sites. Hybrid microassembly techniques combining robotic pick-and-place and capillary self-alignment techniques have been reported to achieve fast transportation and accurate self-alignment [17]. Assembly methods, such as transfer printing and the laser die transfer technique, have also been reported for high-performance transportation of micro components onto the desired assembly sites. Transfer printing is a technique that transfers micro parts using a flexible stamp onto the target substrate [18]. Similar to the transfer printing technique, the laser die transfer technique uses a laser device to release microchips from their carrier and transfer towards a receiving substrate [19]. Both the transfer printing and laser die transfer techniques provide effective solutions for feeding microchips onto receiving receptor sites. However, the transfer printing technique requires a special stamp, and is mainly used for constructing thin structures and stretchable electronics [20,21]. On the other hand, the placement accuracy of laser die transfer is often very low and on the scale of tens of micrometers [19,22]. Moreover, laser die transfer often utilizes a high-power laser source, which may increase the cost of the assembly equipment.

In this paper, we propose a new hybrid microassembly technique to achieve high-throughput and high-accuracy microassembly. The proposed method combines non-contact laser die transfer and parallel mist capillary self-alignment. The laser die transfer technique is used for fast transportation of microchips onto receptor sites and the mist capillary self-assembly technique is applied to achieve parallel and high-accuracy alignment. To transport chips, a low-power and low-cost laser device was employed. Hydrophilic/superhydrophobic patterned surfaces were fabricated and served as receptor sites. Microscopic droplets in the form of water mist were introduced as the media for mist capillary self-alignment. Initial results of this work have been reported in a conference (MARSS 2017) [23]. In addition to the concept and experimental study, this paper reports detailed fabrication methods for the substrate, methods for estimating effective laser burning areas, analysis of the mist-alignment process, related critical volume and forces, and in-depth analysis of the experimental results. 

## 2. Methods and Materials

### 2.1. Materials

**Figure 1:**
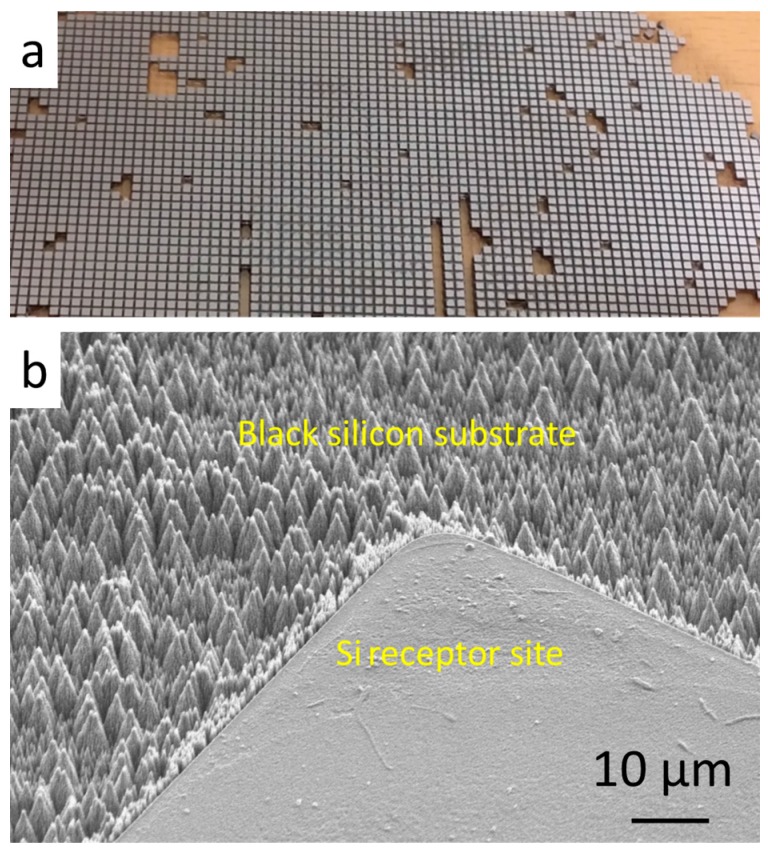
Silicon chips and fabricated receptor sites: (**a**) 500 μm × 500 μm × 300 μm diced silicon chips; and (**b**) 500 μm × 500 μm hydrophilic Si receptor sites with superhydrophobic black silicon substrate.

In this work, microchips were fabricated by dicing 5-inch silicon wafers using a dicing saw (Micro Ace 3, Micro Precision Engineering, Wisconsin, WI, USA) and the size of the diced chips is 500 μm × 500 μm × 300 μm. The diced silicon chips are shown in Figure 1a. The chips were attached to a thermal release tape (Graphene Supermarket, graphene transfer tape) which has similar adhesion as normal adhesive tape at room temperature and loses its adhesive property by heating up to 100 °C. To achieve mist capillary self-alignment, water droplets should be confined inside the receptor sites and a large wetting contrast between the receptor sites and its surrounding is preferred [3]. A similar method [24] was used to fabricate hydrophilic receptor sites and superhydrophobic black silicon substrate, with a different final step where the fluoropolymer coating was removed from the receptor sites using lift-off in buffered hydrofluoric acid. The fabricated patterned surface consists of 500 μm × 500 μm Si pads and black silicon background as shown in Figure 1b. The surface of the black silicon substrate has micro and nano structures. The measured water contact angles are 50° and 170° on Si receptor sites and the superhydrophobic black silicon substrate, respectively. 

### 2.2. Method

**Figure 2:**
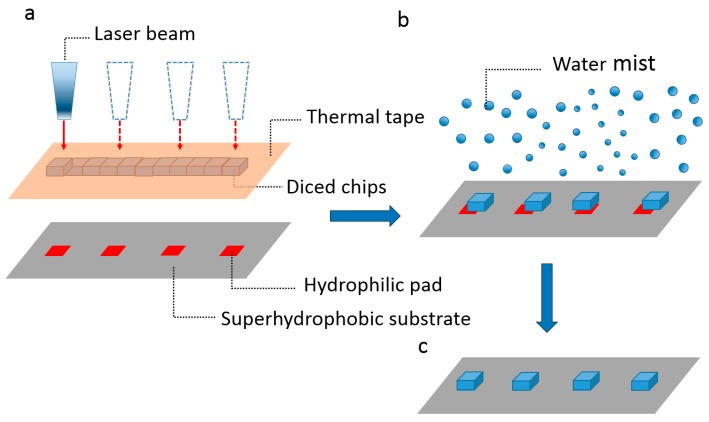
Procedure of laser-assisted mist capillary self-alignment: (**a**) chips were attached to a thermal release tape and a laser beam was pointed at the target chip; (**b**) the laser was fired and the target chips were released from the thermal tape and landed on the receiving receptor sites with an initial placement error; and (**c**) mist-induced micro droplets were delivered to the assembly sites and microchips were aligned to the corresponding receptor sites.

We propose a laser-assisted mist capillary self-alignment method, which combines laser die transfer technique and mist capillary self-alignment. The proposed method is illustrated in Figure 2, the microchips were firstly attached to a thermal release tape (Figure 2a); next the laser beam was pointed at the target chips and the laser fired; then the chips were released from the tape and landed on the corresponding receptor sites with an initial placement error when the curing temperature of the thermal tape was reached, as shown in Figure 2b. Next, water mist was introduced to the assembly sites. Finally, all the chips were self-aligned with the corresponding receptor sites.

### 2.3. Experimental Setup

A robotic system has been set up for carrying out the laser die transfer experiments. The system consists of a laser device (Wicked Lasers, EVO, Euro Intlchoice Tech. Ltd), a sample carrier mounting on three motorized stages (Physik Instrumente M-122.2DD, M-404.8PD, and M-111.1DG, PI GmbH & Co. KG, Karlsruhe, Germany) and two microscopes (InfiniTube and VZM 1000i, Edmund Optics, Barrington, NJ, USA) connected to two identical charge-coupled device (CCD) cameras, respectively (IGV-B1621, Imprex, Milwaukee, WI, USA). In our tests, we have selected a low-cost and low-power laser device with a wavelength of 532 nm and a power of 100 mW. The laser operates in continuous mode. A focal lens was attached to the laser device to release the microchips from the thermal release tape. The motorized stages were used to move the test samples along three axis. The CCD cameras were used to calibrate the coordinates of each microchip relative to the laser beam coordinates and record the releasing process of microchips. Due to the high brightness of the laser beam, a protective glass was placed in front of the side view microscope to protect the reflected laser light damaging the side view CCD camera. Mist capillary self-alignment was carried out using an ultrasonic humidifier (model: Bionaire Ultrasonic Compact BU1300W-I, Bionaire Inc., Montreal, QC, Canada), which can generate a massive amount of microscopic droplets. To spread the mist evenly on the sample, two silicon tubes were connected to the exit of the humidifier and placed near to the sample carrier from two opposite directions. 

## 3. Results

### 3.1. Laser Beam Diameter Estimation

**Figure 3:**
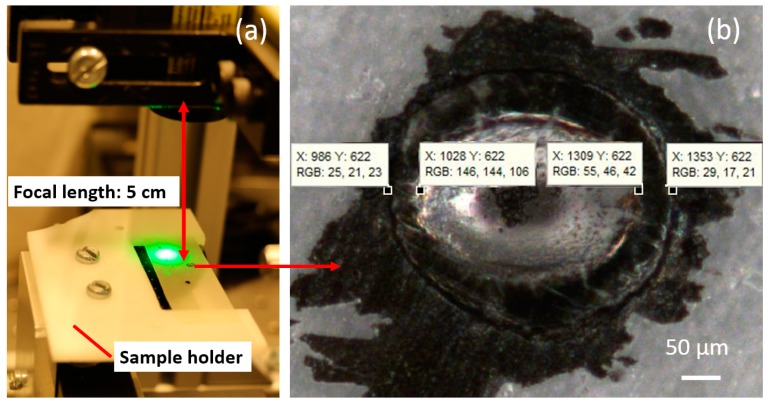
Laser beam diameter estimation: (**a**) the tape is mounted roughly 5 cm away from the focal lens and a reference mark is used to estimate the laser beam diameter at its maximum intensity; and (**b**) zoomed image of a laser beam burn with the inner and outer diameter in pixels.

**Figure 4:**
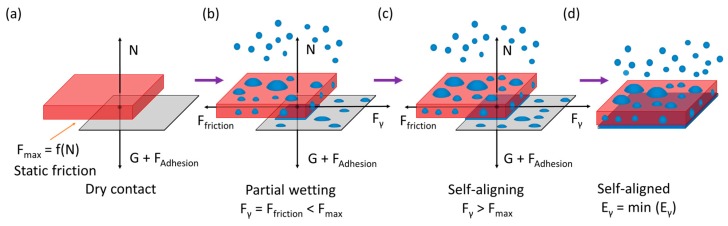
Schematic and force configuration of mist capillary self-alignment process: (**a**) a chip is placed on the top of a receptor site and dry contact forms in between; (**b**) mist induced droplets are delivered onto the assembly site and partially wet the gap between the chip and receptor site; (**c**) droplets wet the whole gap and overcome the static friction force while driving the chip to self-align with the receptor site; and (**d**) the surface energy of the meniscus in the gap is minimized and the chip is aligned with the receptor site.

A stable release of a microchip from a thermal tape requires a powerful enough laser device, a large enough laser beam diameter, and a uniform laser beam intensity distribution [25]. Ideally, the laser beam should cover the whole surface area of the wafer die at the laser beam focal point. An estimation of the laser beam diameter was performed as shown in Figure 3. To further increase the burning power, a focal lens was attached to the laser device and the focal length is roughly 5 cm, as indicated in Figure 3a. A piece of thermal release tape was attached to the sample holder and one reference mark was marked on the thermal release tape. The sample holder was mounted on the sample carrier and moved roughly 5 cm from the focal lens. The test was repeated three times, where the reference mark was burned with the focused laser beam for 2 s. After the reference mark was burned with laser, the burn mark was inspected and calibrated with a microscope (SZ-STU2, Olympus, Tokyo, Japan) as shown in Figure 4b. 

The inner and outer diameters of the laser beam burn marks are presented in micrometers, as shown in Table 1. The inner diameter represents presumably the diameter of the laser beam when its intensity is at its maximum. This estimation was done to obtain an idea of the scale of the laser beam diameters and it was used to narrow down the size of microchips used in die release tests.

### 3.2. Mist Capillary Self-Alignment Process

Mist capillary self-alignment is based on the principle of surface energy minimization, where the gradient of potential drives the chip to align with the receptor site. In our case, the alignment process involves drops merging and liquid meniscus formation. The schematic and force configuration of the mist capillary self-alignment process is shown in Figure 4, in which *G*, *N*, *F_max_*, *F_Adhesion_*, *F_friction_*, *F_γ_* and *E_γ_* represents the gravity of the chip, normal force, static friction force, adhesion force, friction force, surface tension force, and surface energy, respectively. 

**Figure 5:**
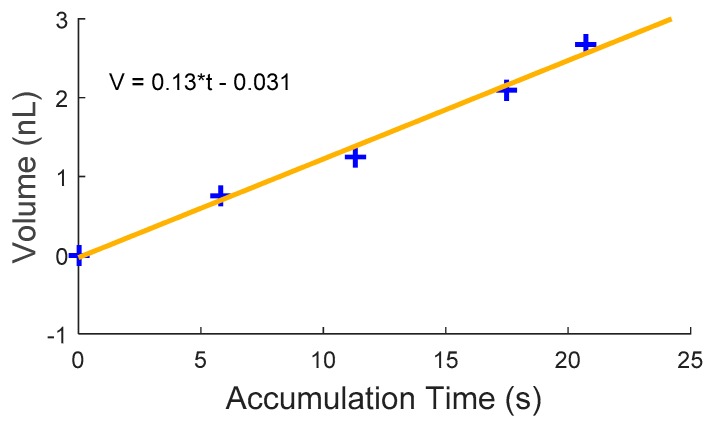
Linear water accumulation process.

Firstly, a chip is transferred onto a receptor site using the laser die transfer technique and the chip is in dry contact with the receptor site, the normal force *N* equals the sum of the gravity *G* and adhesion force *F_Adhesion_*, and the magnitude of the static friction force depends on the normal force (Figure 4a). Then water mist is delivered to the assembly site and droplets start to accumulate and merge with each other on both the chip and the receptor site; gradually, a water meniscus is formed in the gap between the chip and the receptor site and partially wets the gap (Figure 4b). The surface tension of the meniscus *F_γ_* overcomes the dry friction force *F_max_* and pulls the chip towards the receptor site when the volume of the meniscus in the gap increases to a critical volume *V_c_* (Figure 4c). Finally, the chip is aligned to the receptor site where the surface energy of the meniscus *E_γ_* is minimized (Figure 4d). The droplet accumulation process is a linear process [14], where the volume of droplets increase linearly with the accumulation time; therefore, the critical volume *V_c_* to enable self-alignment can be estimated based on the droplet accumulation rate and time on the receptor site. We recorded the droplet accumulation process with a video camera and the volume of the droplets was calculated frame by frame. Figure 5 shows the volume of drops accumulated on a 0.2 mm^2^ receptor site as the function of accumulation time and the relationship can be represented with a linear equation *V* = 0.13*t* − 0.031, where *V* is the volume of the droplets accumulated on the 0.2 mm^2^ receptor site and *t* is the accumulation time. We recorded the size of the gap between the chip and the receptor site and the accumulation time for self-alignment from the captured video (Supplementary Video S1). Based on the accumulation time and the size of the gap, the critical volume for capillary self-alignment can be calculated and estimated, which is about 0.7 nL. 

**Figure 6:**
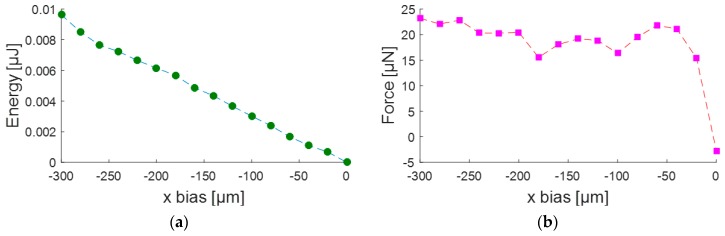
Surface energy (**a**) and restoring force (**b**) as a function of *x* bias.

We simulated the mist capillary self-alignment process for 500 μm × 500 μm × 300 μm chips on the same sized receptor sites using Surface Evolver [26] which finds the static equilibrium for a liquid meniscus by evolving the surface using the gradient descent method. Surface Evolver breaks the surface of the liquid meniscus into smaller elements and minimizes the surface energy of each element by optimizing the location of each vertex. In the simulation, the contact angles of the chip, receptor site, and background were 80°, 50°, 170°, respectively. The volume of the liquid used is the estimated critical volume of 0.7 nL. Figure 6 shows the surface energy and the restoring force versus the misalignment bias. The *x* bias is defined as the placement error between the chip and receptor site along the *x*-axis and the restoring force is the driven force for the chip to align with the receptor site. The results show that the surface energy of the water meniscus is minimized as the *x* bias reaches zero, which is where the chip is aligned with the receptor site. The restoring force is in the range of 15–25 μN which should be in the same scale as the static friction force. 

### 3.3. Laser Die Releasing Procedure

**Figure 7:**
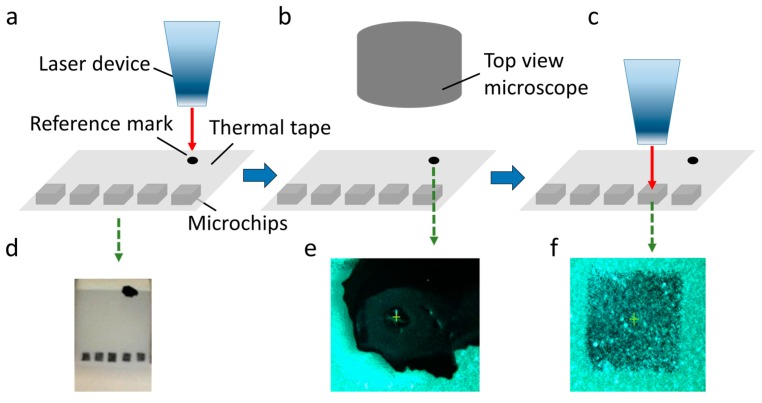
Laser die releasing procedure: (**a**,**d**) a reference mark was moved under the laser beam and the laser was fired at the mark to make a burn; (**b**,**e**) the reference burn mark was moved under a top view microscope and the center of the burn was stored and highlighted with a cross hair; (**c**,**f**) the laser beam focal point was aligned with the center of the chip and the laser was ready to fire to release the chip from the thermal release tape.

We have developed a procedure to carry out laser die releasing tests. The procedure is illustrated in Figure 7: firstly, a black dot was marked as a reference mark on the tape with attached microchips; then, the reference mark was moved under the laser beam and the laser beam was focused at the reference mark to make a burn (Figure 7a,d); meanwhile, the coordinates of the laser beam focal point were stored; after this, the reference mark was moved under the top view microscope and the center of the reference mark burn was stored and highlighted with a crosshair (Figure 7b,e); next, the center of the chip was moved under the top view microscope and their coordinates were stored separately. By subtracting the relative distances between the center of the reference mark burn and the centers of the chips from the previously-stored coordinates of the laser beam focal point, the control script could calculate the correct position of the microchips so that the laser beam focal point is aligned with the center of each chip (Figure 7c,f). These coordinates were stored by the control script and the tape with microchips could be moved to the coordinates by pushing a button on the gamepad. Each of the chips was moved under the laser beam focal point sequentially and the laser beam was fired to release the chip from the thermal release tape. The releasing process was observed and recorded by the side view microscope.

**Figure 8:**
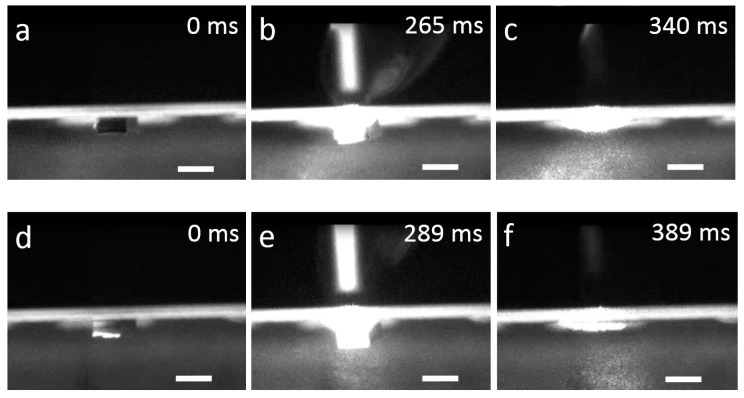
Releasing of chips from a thermal tape by firing a laser beam. (**a**,**d**) 500 μm × 500 μm × 300 μm chips were attached to a thermal release tape; (**b**,**e**) the chips were moved under the laser beam and the laser beam was fired at the chips; (**c**,**f**) the tape was heated to its curing temperature by the laser beam and the chips were released from the thermal release tape (Supplementary Video S2). Scale bar: 500 µm.

We have carried out the die releasing tests using 500 μm × 500 μm × 300 μm silicon chips. The chips were placed manually on a thermal tape and the target receptor sites were mounted between the sample holder and the sample carrier, with the distance between the chips and the receptor sites being about 0.5 mm. Examples of releasing tests are shown in Figure 8, as captured images from Supplementary Video S2. Immediately after the laser was fired, the target area was deformed and the chip was detached from the tape, as shown in Figure 8b,c. The release time was measured to be 340 and 389 ms respectively. The release time of the chip was calculated from the moment the laser was triggered to the moment the chip was fully released from the thermal release tape. The releasing tests were repeated five times, where in each test, the tape with five chips was heated with a continuous laser with power of 100 mW. In total, 25 experiments were performed. Table 2 summarizes the success rate of releasing and the average releasing time from each test, which shows the chips can be released with the releasing time in the range of 300–400 ms. The difference in releasing time may be caused by the following factors: (1) certain randomness due to the material composition and the quality of the initial contact between each chip and the tape; and (2) randomness in the time required to reduce the adhesion force to the threshold of releasing. In a few cases, the chips were partially released where one corner or one edge of the chip was adhered to the thermal tape. This is mainly caused by the misalignment between the laser focal point and the center of the chip. Another cause could be the non-uniform intensity distribution of the laser beam. The reliability of releasing could be improved using a more powerful or larger laser beam with a more uniform spatial laser beam intensity distribution or using a thermal tape with a lower curing temperature. In addition, we also tested 300 μm × 300 μm microchips, which were difficult to release. We attribute the phenomena to two factors: (1) the laser beam causes topographical changes to the thermal tape which leads to similar effective contact area for both 300 μm and 500 μm chips; and (2) the gravity of 500 μm chip is greater, therefore, 500 μm chips are easier to detach.

### 3.4. Mist Capillary Self-Alignment

**Figure 9:**
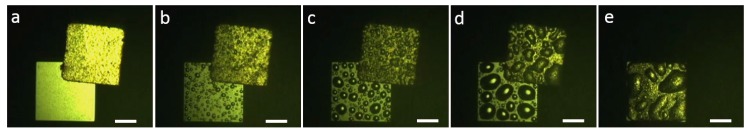
Mist capillary self-alignment: (**a**) a 500 μm × 500 μm × 300 μm chip landed on a receptor site with an initial placement error; (**b**) water mist was delivered to the assembly site; (**c**,**d**) microscopic droplets accumulated at the assembly site; and (**e**) the chip was self-aligned with the corresponding receptor site (Supplementary Video S1). Scale bar: 200 µm.

Immediately after the chips were released from the thermal tape and landed on the corresponding receptor sites, we applied the mist capillary self-alignment method to correct the initial placement error between the chip and the receptor site. Figure 9 shows the typical process of the mist capillary self-alignment. Firstly, a chip landed on the top of a receptor site with an initial placement error, and then water mist was delivered to the assembly site using a commercial ultrasonic humidifier; meanwhile, microscopic droplets accumulated at the assembly site; and, finally, the chip was self-aligned with the receptor site when the volume of the droplets between the gap of the chip and the receptor site increases to the critical volume. Detailed description about the droplets’ accumulation process is described in Section 3.2. We have reported earlier that the alignment accuracy of capillary self-alignment is significantly better than the manufacturing precision of the chips and receptor sites, and the accuracy can be better than 1 μm when the edges of the chips and the receptor sites are well defined [17]. In this work, 10 out of 10 microchips were successfully self-aligned with the corresponding receptor sites, and the alignment accuracy was inspected using an optical microscope. The results show that the alignment accuracy is 1.4 ± 0.8 μm, which is consistent with our earlier results [17]. Mist capillary self-alignment is an entirely parallel process and, in principle, it could be introduced to thousands to millions of assembly sites and realize massive self-alignments simultaneously.

## 4. Conclusions

In this paper, we reported a laser-assisted mist capillary self-alignment method for the assembly of microchips on hydrophilic/superhydrophobic receptor sites. We have demonstrated that laser die transfer can transport microchips from a thermal release tape onto the receptor sites within an accuracy that the mist capillary self-alignment technique can successively apply to achieve high assembly accuracy. Microchips can be released in a non-contact manner from a thermally-curable adhesive tape with a low-cost and low-power commercial laser device. The releasing tests suggest that the release time below 400 ms is achievable using a laser device (100 mW power) for 500 μm × 500 μm × 300 μm silicon chips. In comparison with the hybrid method combining robotic pick-and-place and capillary self-alignment proposed by us earlier, this method is especially suitable for fragile micro components, where non-contact handling is preferred. Furthermore, the method can be potentially applied to ultra-thin chips by employing the laser ablation die transfer technique. The mist capillary self-alignment has been studied through theoretical analysis and experiments. The acting forces for capillary self-alignment have been analyzed and the critical volume enabling capillary self-alignment has been estimated. We have demonstrated that microchips self-align to the corresponding receptor site in parallel with an alignment accuracy of 1.4 ± 0.8 μm. The proposed technique has great potential for high-throughput and high-accuracy assembly of micro devices. Furthermore, since water droplets will eventually evaporate and disappear shortly after the capillary self-alignment, it is possible to integrate the proposed technology with bonding techniques, such as thermal bonding, as reported in our earlier work [27]. The proposed technique can also be applied to microchips containing asymmetric patterns and micro bumps [8]. In future work, more powerful lasers will be investigated to improve the reliability and efficiency of the chips releasing. 

## Figures and Tables

**Table 1:** Inner and outer diameters of the laser beam.

Test	Inner Diameter (μm)	Outer Diameter (μm)
# 1	240.54	316.22
# 2	253.15	330.63
# 3	263.06	315.32
Average	252.58	320.72

**Table 2:** Releasing time and success rate.

Test	Average Releasing Time	Success Rate
# 1	390.3 ms	4/5
# 2	380.4 ms	5/5
# 3	398.1 ms	3/5
# 4	300.2 ms	5/5
# 5	357.3 ms	4/5

## Supplementary Materials

The following are available online at www.mdpi.com/2072-666X/8/12/361/s1, Video S1: Water mist-induced capillary self-alignment, Video S2: Laser die transfer.
